# Background factors for chronic low back pain resistant to cognitive behavioral therapy

**DOI:** 10.1038/s41598-021-87239-2

**Published:** 2021-04-15

**Authors:** Keisuke Shimizu, Kazuhide Inage, Sumihisa Orita, Yawara Eguchi, Yasuhiro Shiga, Masao Koda, Yasuchika Aoki, Toshiaki Kotani, Tsutomu Akazawa, Takeo Furuya, Junichi Nakamura, Hiroshi Takahashi, Miyako Suzuki-Narita, Satoshi Maki, Shigeo Hagiwara, Masahiro Inoue, Masaki Norimoto, Hideyuki Kinoshita, Takashi Sato, Masashi Sato, Keigo Enomoto, Hiromitsu Takaoka, Norichika Mizuki, Takashi Hozumi, Ryuto Tsuchiya, Geundong Kim, Takuma Otagiri, Tomohito Mukaihata, Takahisa Hishiya, Seiji Ohtori

**Affiliations:** 1 Future Medicine Education and Research Organization at Chiba University, Chiba University, 1-8-1 Inohana Chuo-ku, Chiba, 260-8670 Japan; 2 Department of Orthopedic Surgery, Graduate School of Medicine, Chiba University, Chiba, Japan; 3 Center for Frontier Medical Engineering, Chiba University, Chiba, Japan; 4 Department of Orthopedic Surgery, Faculty of Medicine, University of Tsukuba, Tsukuba, Japan; 5 Department of Orthopedic Surgery, Eastern Chiba Medical Center, Chiba, Japan; 6 Department of Orthopedic Surgery, Seirei Sakura Citizen Hospital, Chiba, Japan; 7 Department of Orthopedic Surgery, St. Marianna University School of Medicine, Kawasaki, Japan; 8 Department of Orthopedic Surgery, Toho University Sakura Medical Center, Sakura, Japan; 9 Department of Orthopedic Surgery, Chiba Cancer Center, Chiba, Japan

**Keywords:** Psychology, Diseases, Medical research

## Abstract

This study examined the factors that inhibit the therapeutic effects of cognitive behavioral therapy (CBT) and clarify the adaptation judgment criteria of CBT. We included patients with chronic low back pain and allocated them to the adaptation (with visual analog scale [VAS] improvement) or non-adaptation group (without VAS improvement). The patients were analyzed using various psychological tests. CBT improved depressive symptoms and catastrophic thinking; however, they were not correlated with the VAS and did not directly affect low back pain improvement. The non-adaptation group showed an unexplainable/vague sense of anxiety; an excessive focus on searching for pain; a strong intimacy desire; a strong tendency of medical dependency; and fantasy or distortion of the actual experience, especially self-image. Moreover, the patients showed a low ability to objectively express or attribute meaning to pain due to poor language skills, attention-deficit hyperactivity disorder, and emotional value judgment. Individuals with the aforementioned characteristics of pre-CBT psychological tests should select a different treatment approach given the high poor-adaption possibility. Even patients with depressive or anxious symptoms are not necessarily adaptable for CBT. Therefore, pre-CBT tests for treatment suitability are necessary. Future studies should establish a protocol for psychotherapy suitable for the non-adaptation group.

## Introduction

In Japan, the lifetime prevalence of low back pain is 83%^1^ with > 75% and ≈ 22% of low back pain cases having apparent and unknown causes, respectively^2^. A study in the US reported that chronic low back pain resulted in the loss of an average of 4.6 working hours/week^3^. In Japan, a corresponding loss of working hours could result in a significant economic loss of approximately 189 million yen^4^. Given that both organic and psychosocial factors could be involved in non-specific cases of low back pain, cognitive behavioral therapy (CBT), which focuses on psychosocial factors, has been performed with a certain level of evidence^5^. However, CBT has a limited/short analgesic effect and a significant effect on emotion and life disorder^6^; therefore, CBT might have a limited effect on pain despite its pain-relief expectation.

Based on these findings, several randomized controlled trials have selected the quality of life, life obstacle, and emotional issues, rather than “pain relief”, as primary endpoints. Even the recent well-practiced acceptance and commitment therapy and mindfulness-based stress reduction (collectively known as third-generation CBT) have a greater focus on facilitating the pursuit of valuable goals/purposes with pain tolerance, rather than pain reduction. Since there is no significant difference in pain relief between the third-generation and conventional CBT^7^, direct pain reduction using the current CBT might be difficult. Although several patients receive CBT through the outpatient referral system as a recommended therapy in the Japanese treatment guidelines for low back pain based on the aforementioned limited evidence^5^, the therapeutic adaptation of CBT is at the discretion of the doctor or clinical psychotherapist since there are no CBT guidelines. Consequently, there are many cases where patients with no response to CBT are overlooked or continue undergoing CBT due to a lack of alternative treatments. Additionally, although CBT practice requires specific expertise and extended periods, it is still not covered by the national insurance. Taken together, although CBT has a high-cost burden on patients, it has low cost-effectiveness for pain relief.

However, some patients show a significant pain reduction with CBT; therefore, there is a need to examine background factors underlying the presence/absence of a CBT response. Several studies have reported psychosocial factors as a background factor in patients with chronic low back pain; moreover, the revised definition of chronic low back pain by “The International Society for the Study of the Lumbar Spine”^8^ describes pain as an emotional experience. Yet, the presence of psychosocial factors as a background factor for chronic low back pain are not related to suitability of CBT treatment directly. Although there have been meta-analysis studies on CBT for chronic low back pain^9, 10^ and reports of psychosocial factors contributing to an improved treatment effect on low back pain^11^, there has been no discussion regarding background factors in patients without a response to CBT. Additionally, a study that used the Rorschach test to assess the psychological characteristics of patients with chronic pain (not limited to low back pain)^12^ reported that “it tended to be a poorly flexible and more pessimistic view, strong interest in one’s own body; however, it may be difficult to adjust emotional regulation due to the easy perception of aggression as being slightly defensive in interpersonal relationships”. Given that this is also generally true for patients with chronic pain, it might not be a unique characteristic of patients with chronic low back pain who are resistant to CBT.

As aforementioned, previous studies have mainly assessed common characteristics among patients with chronic low back pain or background factors that promote CBT treatment effects; however, there has been no study on background factors with negative therapeutic effects. This study aimed to clearly define the target patient group for CBT by identifying psychosocial factors that impede the CBT treatment effects on pain reduction.

## Materials and methods

### Study participants

The study included 46 patients with chronic low back pain without a surgery history for low back pain who have been referred to the center for outpatients of orthopedic spine surgery and pain at our institution since April 2018. Using the Brief Scale for Psychiatric Problems in Orthopedic Patient [physician and patient versions] (BS-POP), which is a simple questionnaire that enables the evaluation of psychiatric problems in orthopedic patients^13^, four spinal surgeons determined there was a strong possibility that psychosocial factors contributed to the experiences of these patients. In addition, based on MRI scans and neurological symptoms, the physicians at our institute determined that these patients lacked primary organic factors (that met diagnostic criteria) caused by low back pain symptoms. Furthermore, at the intractable case conference, eight senior physicians confirmed that the MRI findings did not match the patient’s symptoms. The patients presented resistance to orthopedic treatments, including medication, exercise therapy, and various block injections. Consequently, standard orthopedic treatment was continued during CBT intervention without altering oral administration.

Our study was performed in accordance with the Declaration of Helsinki regarding the ethical principles for medical research involving human subjects. The study protocol was approved by the Chiba University Ethics Committee. All experiments were performed in accordance with these guidelines and regulations. Following provision of a complete description of the study to the all patients, written informed consent was obtained prior to study initiation.

### Test item

The included patients underwent assessments using the following tools: Visual Analog Scale (VAS), Japanese version-Hospital Anxiety and Depression Scale (HADS)^14,15^, Japanese version-Pain Catastrophic Scale (PCS)^16,17^, Autism-Spectrum Quotient (AQ)^18,19^, and Adult ADHD Self-Report Scale-V (Translated in Japanese by the author) (ASRS)^20^. Moreover, individual cases underwent assessments using the Wechsler Adult Intelligence Scale-IV (WAIS-IV)^21–24^ and Rorschach Test^25^ on different days.

### CBT

Two certified public psychologists (clinical psychologists) with over 10 years of experience conducted CBT. The adopted CBT techniques applied psychoeducation, pacing, relaxation (abdominal breathing, progressive muscle relaxation), automatic thought, distraction, cognitive restructuring, and behavioral activation^26,27^. Given the limited allocation for reservations, 10 sessions (50 min/session) were held every two weeks. The details of the CBT session are shown in Table 1.

**Table 1:** Protocol of CBT.

Session	Program	Contents
1	Psychoeducation1	Theory of biopsychosocial model
2	Psychoeducation2	Brain function related to pain
3	Pacing	How to accomplish tasks in a thoughtful and sensible way
4	Relaxation training	Techniques to decrease stress and muscle tension, including homework
5	automatic thought	Understand the thought that person has automatically response to pain^a^
6	Distraction	Distract and draw attention away from pain
7	Cognitive restructuring 1	Identify unhelpful thought and increase balanced thinking^a^
8	Cognitive restructuring 2	Identify unhelpful thought and increase balanced thinking^a^
9	Behavioral activation	Increase engagement in rewarding and meaningful activities
10	Review	Reviewing all CBT program, question and answer session

^a^Including homework.

### Statistical analyses

Statistical analyses were performed using IBM SPSS Statistics 23 (IBM, Armonk, NY, USA). Between-group differences for each variable in the HADS, AQ, ASRS, WAIS-IV, and Rorschach tests were determined using the U-test with no assumption of normal distribution and with consideration of dispersive deflection. Regarding EB (experience balance), SumT (needs and openness to close emotional relations), SumV (tendency to focus less on their positive sides), food response (index of dependence property), W:M (level of desire for achievement) in the Rorschach test, the frequency was evaluated using the chi-square test given the clear difference in the interpretive hypothesis according to the score.

## Results

### Preliminary analyses

Using the improvement criteria shown in Table 2^28^, the pre- and post-CBT VAS values were evaluated using five levels (marked improvement, moderate improvement, mild improvement, unchanged, and worse), and patients were divided into the adaptation group (showing mild improvement or better) and non-adaptation group (unchanged or worse). The patients answered the average value of the VAS score for the previous week both before and after CBT. As a result, 18 patients were assigned to the adaptation group, while 28 patients were assigned to the non-adaptation group. The diagnosis results, duration of pain complaint, and pain medications taken are shown in Table 3.

**Table 2:** Improvement criteria table about VAS value.

		VAS value (post intervention)										
		0 ~ 4	5 ~ 14	15 ~ 24	25 ~ 34	35 ~ 44	45 ~ 54	55 ~ 64	65 ~ 74	75 ~ 84	85 ~ 94	95 ~ 100
VAS value (pre intervention)	25 ~ 34	1	2	2	3	4	4	5	5	5	5	5
	35 ~ 44	1	2	2	3	4	4	5	5	5	5	5
	45 ~ 54	1	1	2	2	3	4	4	5	5	5	5
	55 ~ 64	1	1	2	2	3	4	4	5	5	5	5
	65 ~ 74	1	1	1	2	2	3	4	4	5	5	5
	75 ~ 84	1	1	1	2	2	3	4	4	5	5	5
	85 ~ 94	1	1	1	1	2	2	3	4	4	5	5
	95 ~ 100	1	1	1	1	2	2	3	4	4	5	5

1. Very much improved, 2. Much improved, 3. Minimally improved, 4. Static, 5. Worsen.

**Table 3:** Demographic characteristics of study participants.

	Diagnosis	
	Adaptations	Non-adaptatons
None	12	18
Mild disc degeneration	3	6
Mild idiopathic scoliosis	1	2
Mild spondylolisthesis	2	2

	Durations	
	Adaptations	Non-adaptatons
6 ~ 12 month	2	4
1 ~ 2 years	10	12
3 ~ 4 years	5	8
Over 5 years	1	4

	Medications(total number)	
	Adaptations	Non-adaptatons
None	2	5
NSAIDs	8	10
Acetaminophen	4	11
Pregabalin/mirogabalin	3	4
Tramadol hydrochloride	3	3
Duloxetine	1	2

	Job	
	Adaptations	Non-adaptatons
Employed	10	12
Unemployed	8	16

	Househeld	
	Adaptations	Non-adaptatons
Alone	6	8
With family	12	20

### Post-CBT VAS scores for low back pain

**Figure 1:**
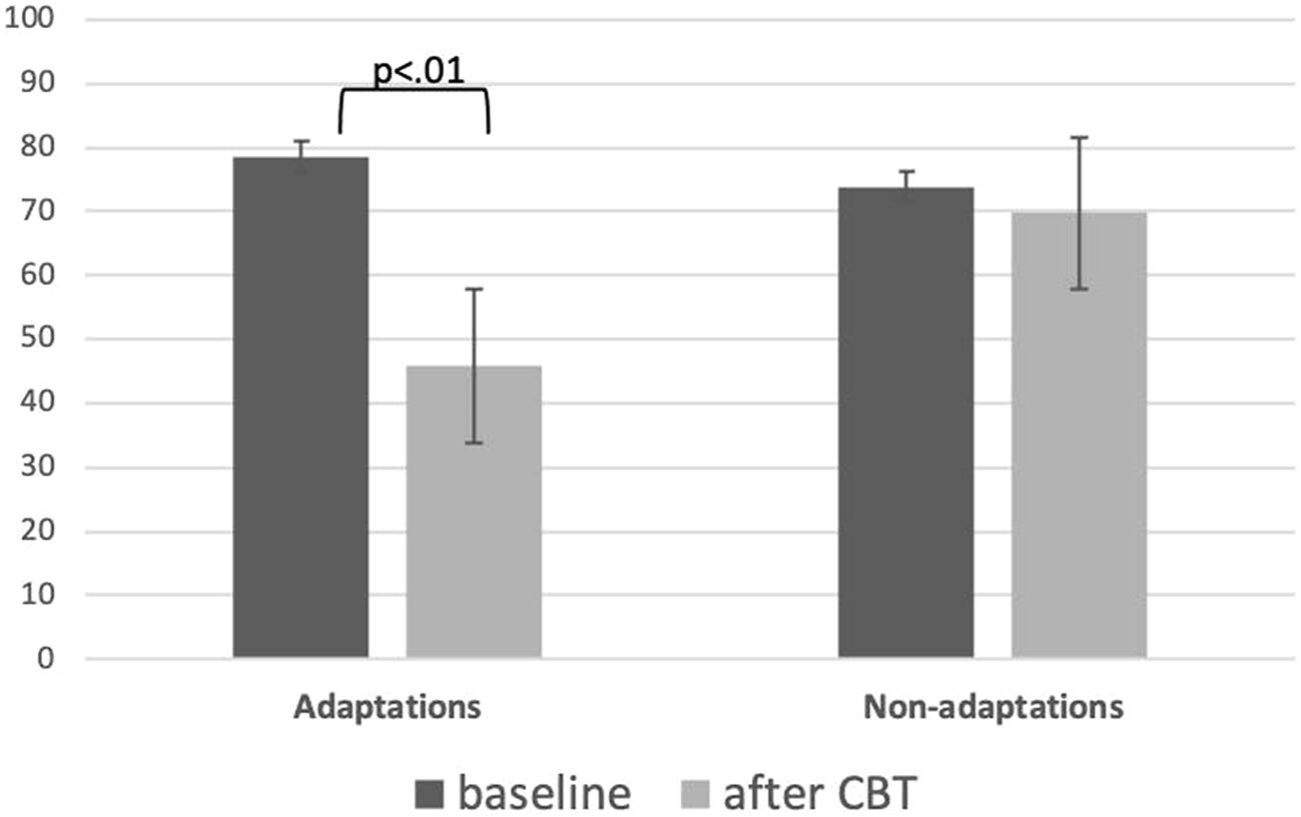
VAS value after the CBT intervention. The U-test was used to analyze the average difference in pre- and post-CBT VAS score for low back pain. After 10 sessions, there were 18 patients with improvement in the VAS score for low back pain (78.50 ± 15.31 → 45.87 ± 16.40). Further, there were 28 patients without improvement in the pain VAS score (73.80 ± 17.78 → 69.70 ± 16.78). Thus, we confirmed a VAS score improvement in 39.13% of all patients.

As shown in Table 4 and Fig. 1, the 18 patients in the adaptation group ( VAS score improvement from 78.50 ± 15.31 to 45.87 ± 16.40), while the 28 patients in the non-adaptation group (VAS: 73.80 ± 17.78 → 69.70 ± 16.87). Therefore, 39.13% of the included patients presented improvement in low back pain. Sex differences and VAS scores were not significantly different and did not affect the adaptations and non-adaptations grouping.

**Table 4:** VAS value after the CBT intervention.

Sex: male/female(age ± SD)	Adaptations (n = 18)		Non-adaptations (n = 28)		*p*
	10 m/8 f (43 ± 11.51)		10 m/18 f (55 ± 16.07)		
Scale	Average	SD	Average	SD	
VAS(baseline)	78.50	15.32	73.80	17.78	0.49
VAS(after CBT)	45.88	16.40	69.70	16.87	0.00

### Examination of the difference in the average AQ and ASRS scores

Table 5 presents the results. There was no significant difference in the AQ scores between the adaptation group (16.00 ± 6.86) and the non-adaptation group (17.80 ± 11.96). Additionally, there was an autism tendency with the lower average results. Contrastingly, the non-adaptation group had a significantly higher ASRS score (14.90 ± 2.79) than the adaptation group (8.13 ± 5.08; p < 0.01). This indicated that the non-adaptation group had a stronger tendency for ADHD.

**Table 5:** Examination for a difference in average value of AQ and ASRS.

Scale	Adaptations		Non-adaptations		*p*
	Average	SD	Average	SD	
AQ	16.00	6.86	17.80	11.96	0.62
ASRS	8.13	5.08	14.90	2.79	0.01

### Examination of the intelligence level

**Figure 2:**
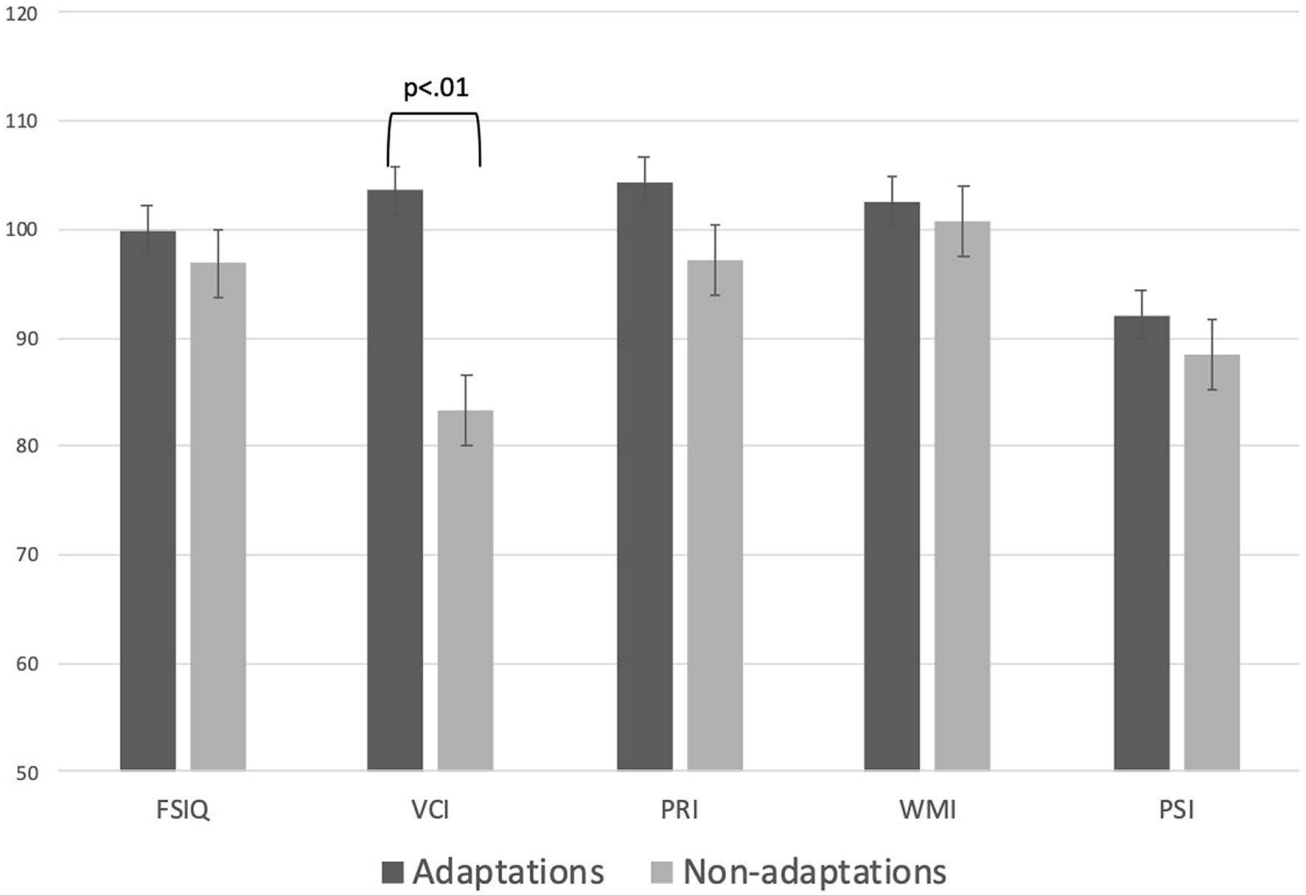
Examination for intelligence level. The WAIS-IV results in each group are shown. There were no significant between-group difference in overall test IQ, perceptual reasoning, working memory, and processing speed, which were indicative of an average3 intellectual level. However, in the non-adaption group, the verbal comprehension index (83.35 [± 11.70]) was determined as a dull normal level compared with the same-age segment; moreover, there was a significant between-group difference (p < 0.01). Compared with the adaptation group, the non-adaptation group may have significantly lower verbal comprehension (i.e., language communicating ability to understand or express the language).

Table 6 and Fig. 2 present the WAIS-IV results. There was no significant between-group difference in the overall IQ test, perceptual reasoning, working memory, and processing speed; moreover, both groups showed scores indicating an average intellectual level. However, comparisons of the scores within the same age group, only the average verbal comprehension index (VCI) in the non-adaptation group was slightly lower than the normal level; moreover, there was a significant between-group difference (p < 0.01). VCI assesses an individual’s comprehension skills, difficulties with new/unexpected situations, preferences for verbal information, and ability to draw upon learned information, and reason through answers/express thoughts. Between-group comparisons revealed significantly lower VCI in the non-adaptation group.

**Table 6:** Examination for intelligence level.

	Adaptations		Non-adaptations		U-test
	Average	SD	Average	SD	
FSIQ (full scale intelligence quotient)	99.88	11.87	96.85	14.90	0.63
VCI (verbal comprehension index)	103.63	14.12	83.35	11.70	0.00
PRI (perceptual reasoning index)	104.38	6.16	97.10	13.20	0.16
WMI (working memory index)	102.63	11.17	100.75	13.04	0.73
PSI (processing speed index)	92.13	8.15	88.50	15.31	0.55

### Examination for average differences in the pre- and post-CBT HADS and PCS scores .

Table 7 presents the findings. In the HADS (HADS is not a test for diagnosing depression or anxiety disorders, but a test for measuring depressive and anxious symptoms), the baseline score for the anxiety scale/depression scale was 9.38 ± 2.34/9.13 ± 2.57 and 8.15 ± 2.92/7.45 ± 3.71 in the adaptation and non-adaptation groups, respectively, with no significant between-group difference. Based on their high average values, both groups were determined as “Suspicious” for both depression and anxiety. The post-CBT scores for the anxiety scale/depression scale were 4.12 ± 1.61/4.62 ± 1.57 and 7.40 ± 3.12/4.25 ± 3.20 in the adaptation and non-adaptation groups, respectively. This indicated a significant improvement in the scores for both anxiety and depression scales in the adaptation group, but only the depression scale in the non-adaptation group (p < 0.01). There was a moderate correlation of the VAS score with the anxiety scale (r = 0.51) but not the depression scale (r = 0.23).

**Table 7:** Average defference in HADS and PCS before/after the CBT intervetion and R with VAS value.

	Adaptations		*p*	Non-adaptations		*p*	R with VAS value
	Baseline	After CBT		Baseline	After CBT		
PCS	32.25(± 8.73)	19.5(± 5.78)	0.00	32.30(± 10.07)	21.55(± 9.89)	0.00	0.26
HADS(anxiety)	9.37(± 2.34)	4.12(± 1.61)	0.83	8.15(± 2.95)	7.40(± 3.12)	0.45	0.51
HADS(depression)	9.12(± 2.57)	4.62(± 1.57)	0.00	7.45(± 3.70)	4.25(± 3.20)	0.01	0.23

The baseline PCS scores indicated a high level (recognition of catastrophic thinking) in both the adaptation (32.25 ± 8.73) and non-adaptation groups (32.30 ± 10.07). Further, there was a significant post-CBT improvement in the PCS score in both the adaptation (19.5 ± 5.78) and non-adaptation groups (21.55 ± 9.89) (p < 0.01). The VAS score was extremely weakly correlated with the PSC score (correlation coefficient = 0.26) as an extremely weak correlation; however, it was difficult to determine whether this was a significant correlation coefficient for the treatment.

### Rorschach index

#### Common factor for both groups

Tables 8 and 9 present the results. First, given that the D Score (stress tolerance and elements of control) indicates “− or negative” as a common factor for both groups, there could be impulsive decision making due to the lack of internal control with the stimulation overload condition. Since they lack a consistent approach for problem solving and decision making due to the frequent Ambitent (indeterminate) form (p < 0.01), they are most likely to make decisions on a day-to-day basis given the lack of improvement through a learning experience in a trial and error process. Additionally, W:M indicated self-desire for a significantly high achievement level, as well as a significantly high positive rate in all cases (p < 0.01). Since the patients tended to set goals that exceeded their ability, they may easily experience failure or setbacks.

**Table 8:** Χ^2^ test for coping style, SumV, SumT, food, W:M.

		Frequency		Χ^2^ Test	All cases	Χ^2^Test
		Adaptations	Non-adaptations			
Coping style	Extratensive	4	6	0.12	17	0.01
	Introversive	4	3	0.31		
	Ambitent	10	19	0.29	29	
SumV	1 < V	5	19	0.02		
	1 > V	13	9	0.53		
SumT	T > 1	4	16	0.02		
	T = 1	4	4	0.32		
	T = 0	10	8	0.24		
Food	Food = 0	12	10	0.31		
	Food > 1	6	18	0.01		
W: M	Positive	14	21	0.31	22	0.01
	Negative	4	7	0.29	6

**Table 9:** Rorschach test index.

		Adaptations		Non-adaptations		U-test
		Average	SD	Average	SD	
Control	R	19.00	3.28	24.80	7.72	0.06
	lambda	0.53	0.27	0.62	0.42	0.75
	EA	6.06	3.57	7.70	3.51	0.15
	es	8.13	3.14	11.75	6.92	0.30
	D	− 0.50	1.58	− 1.45	2.40	0.50
	AdjD	− 0.50	1.58	− 1.15	2.24	0.71
	FM	4.25	1.48	5.80	3.75	0.44
	m	0.50	0.71	0.40	0.66	0.78
	SumC'	1.50	1.58	2.50	2.29	0.35
	SumV	0.25	0.43	1.25	1.17	0.04
	SumT	0.75	1.09	1.65	1.88	0.28
	SumY	0.88	1.27	1.65	2.48	0.78
Affect	FC	3.73	0.97	2.55	2.36	0.67
	CF + C	1.55	0.97	1.80	1.47	0.50
	PureC	0.13	0.33	0.45	0.86	0.57
	SumC'	2.55	2.25	2.55	2.25	0.28
	WSumC	1.50	1.58	3.30	1.97	0.33
	Afr	0.51	0.14	0.57	0.21	0.57
	S	1.00	1.00	1.95	1.80	0.28
	Blends	7.75	3.56	4.65	3.41	0.05
	CP	0.00	0.00	0.05	0.22	0.86
Inter personal	COP	1.75	2.05	0.85	1.06	0.41
	GHR	4.00	2.74	4.10	1.89	0.64
	PHR	1.63	1.49	3.30	2.05	0.05
	a	4.63	2.83	5.80	3.17	0.35
	p	4.50	2.50	5.20	3.40	0.47
	Food	0.38	0.70	1.00	1.32	0.04
	Human content	5.00	3.74	5.75	2.02	0.20
	Pure H	3.50	3.04	2.50	1.66	0.22
	PER	0.75	1.39	1.75	1.87	0.98
	Isolation index	0.08	0.07	0.11	0.15	0.50
	AG	0.38	0.48	0.50	0.81	0.35
Ideation	Ma	2.00	2.74	1.90	1.37	0.44
	Mp	2.13	1.69	2.75	1.30	0.15
	2Ab + Art + AY	1.00	1.22	1.75	1.97	0.14
	MOR	0.75	1.09	1.65	1.53	0.86
	Sum6	1.88	1.17	3.30	2.28	0.61
	Level2	0.00	0.00	0.05	0.22	0.11
	Wsum6	5.00	3.91	9.75	7.30	0.47
	M−	0.13	0.33	0.40	0.66	1.00
	Mnone	0.00	0.00	0.00	0.00	0.12
Mediation	XA%	0.91	0.07	0.87	0.08	0.14
	WDA%	0.91	0.07	0.87	0.07	0.09
	X−%	0.09	0.07	0.14	0.07	0.60
	S-	0.13	0.33	0.30	0.56	0.60
	P	4.63	1.49	5.10	1.89	0.03
	(P)	1.00	0.50	0.40	0.58	0.11
	X + %	0.74	0.11	0.64	0.12	0.20
	Xu%	0.17	0.10	0.23	0.10	0.78
Processing	Zf	13.25	3.56	13.65	5.22	1.00
	W	9.75	3.15	10.00	4.84	0.10
	D	7.50	2.87	11.40	5.74	0.35
	Dd	1.75	1.09	4.10	3.50	0.24
	M	3.88	3.33	4.40	1.90	1.00
	Zd	− 2.38	4.37	− 2.08	6.32	0.64
	PSV	0.38	0.48	1.00	2.30	0.86
	DQ +	6.50	2.60	6.25	3.59	0.24
	DQv	0.75	1.09	1.75	1.87	0.20
Self perception	3r + (2)/R	0.33	0.17	0.41	0.12	0.86
	Fr + rF	0.00	0.00	0.05	0.22	0.53
	FD	1.37	0.43	0.85	1.22	0.06
	An + Xy	0.50	0.71	2.00	1.67	0.03
	H	3.63	2.96	2.50	1.66	0.33
	(H) + Hd;(Hd)	1.75	0.83	3.25	1.73	0.02
Special indices	S-CON	2.75	0.83	4.15	1.68	0.02
	PTI	0.00	0.00	0.45	0.67	0.17
	DEPI	3.00	0.87	4.15	1.19	0.03
	CDI	2.75	0.83	3.15	0.91	0.35
	HVI	No		No		
	OBS	No		Yes1		

#### Unique factor in the non-adaptation group

##### Control and stress tolerance

An unexplainable/vague sense of anxiety occurs because SumY (situational stress-related psychological helplessness) is thrice larger than “m(interpersonal conflict)”. Additionally, given that SumV > 1, it focuses on excessive self-monitoring behavior (pain scrutiny) for self-negative aspects (p < 0.01). Further, it represents a lack of psychological sophistication due to fewer blend responses (4.65 ± 3.40). Therefore, this could be an emotional behavior rather than a behavior allowing sufficient objective determination of pain.

##### Self-perception

In the adaptation group, the value for [H:(H) + Hd + (Hd)] (ability of reality-based perception of self and others) was 3.65:1.75; the self-image was mainly formed based on actual experience rather than imagination. In the non-adaptation group, the value for [H:(H) + Hd + (Hd)] was 2.5:3.25 and the self-image tended to be formed based on distorted actual experience or imagination.

##### Interpersonal perception and behavior

Given that SumT > 1 and Food > 1, it was indicative of a very strong desire for intimate relationships (P < 0.01).

##### Affect

The adaptation group showed a tendency of suppression or excessive control of emotional confusion, as indicated by W SumC (overt reactivity of feelings) < SumC’ (excessive internalization of feelings) and FC (emotional expression) = 3.73 (± 0.96) or 2.4 times larger than CF + C (controlled emotion). On the other hand, the non-adaptation group showed a tendency of expressing confusion with mild emotional adjustment, as indicated by WSum C > SumC’ and CF + C = FC.

##### Information processing

The score of Dd (focus more on minute ore unusual features of a new field of information with more processing effort) > 4 indicated a tendency to focus on insignificant things rather than essential things. Additionally, it indicated a tendency to prefer non-complicated stimuli and to extensively simplify things for their understanding.

## Discussion

Several studies have assessed the relationship between chronic low back pain and psychosocial factor; moreover, CBT has been recommended given the high prevalence of depression, anxiety disorder^29^, or ASD/ADHD^30, 31^. However, given that CBT does not improve low back pain in many cases, this study aimed to comprehensively evaluate and clarify the psychosocial factors that negatively affect CBT treatment.

In the non-adaptation group, the presence of ADHD tendency with distractibility, hyperactivity, and impulsivity as background factors may impede successful CBT implementation. Autism tendency was considered to have a weak relationship with chronic low back pain due to the low AQ score. The WAIS-IV scores revealed that the overall IQ was maintained in both groups; however, the verbal IQ (83.35) was lower in the non-adaptation group than in the same age group and adaptation group. It may have been difficult for the non-adaptation group to learn the relevant CBT skills due to lack of knowledge, poor comprehension, or biased interests.

As aforementioned, both groups had a sense of depressive and anxious symptoms; however, the non-adaptation group did not show a post-CBT improvement in the sense of anxious symptoms. According to the results of Rorschach test, the non-adaptation group’s sense of anxiety was vague, therefore this anxiety should be clarified in psychotherapy before psychiatric treatment is recommended. Additionally, there was no correlation between depressive symptoms/catastrophic thinking and VAS scores. Since the non-adaptation group remained even after confirmed improvement in these parameters, they could have a poor direct relation with the improvement of low back pain. In general, depressive symptoms and catastrophic thinking have been considered as factors that increase pain intensity; however, having a vague sense of anxiety may also contribute to pain. In the future, it is necessary to investigate the factors that increase pain in more detail.

Regarding common psychological factors for patients with chronic low back pain shown in the Rorschach Test, there are three main points: (1) patients become more emotional as they lose objectivity of decision-making and value judgment, (2) there is no plan for solving problems with a consistent approach, and (3) it is easy to experience failure or setback when demanding an achievement standard beyond one’s ability. These factors could be improved using CBT.

Contrastingly, regarding unique factors in the non-adaptation group, there are the following four points: patients had (1) an unexplainable/vague sense of anxiety ((2) an excessive search for pain, (3) extremely high dependency needs, and (4) a tendency to form a self-image based on fantasy and distorted actual experience. From all other psychological test, this finding may be attributed to a lack of objective thinking process, low VCI, and high impulsivity due to ADHD tendency. Consequently, it might be difficult to manage low back pain in patients with these characteristics, using CBT. When considering the indications for CBT, it is therefore necessary to carefully consider whether patients with chronic low back pain have these characteristics.

This study has several limitations, including the limited number of treatment practices (10 times) due to time constraints, the lack of diagnoses of psychiatric or developmental disorders due to lack of an intervention by a psychiatrist, and the small number of participants (46 patients). Furthermore, it is possible that the effects of CBT were not be accurately measured due to the lack of a control group who did not undergo CBT, and the lack of consideration of confounding variables in the home and work environment. In the future, we intend to establish optimal psychotherapy protocols for the non-adaptation group, as well as to set guidelines to decide whether CBT is appropriate for patients by establishing evidence using larger sample sizes.

## Conclusion

Our findings revealed the following unique factors in the non-adaptation group: a vague sense of anxiety, emotional searching behavior for pain, tendency to form a pain perspective based on fantasy or distorted actual experience, excessive desire for a close relationship, and strong medical care dependency. This may be attributed to a low verbal comprehension ability and ADHD tendency. A different treatment approach may be desirable for patients with the aforementioned characteristics upon pre-CBT psychological examination. Therefore, there is a need for early-stage identification of CBT inhibitors and subsequent determination of whether the patient is suitable to undergo CBT. The present findings could provide a basis for the establishment of a psychotherapy protocol suitable for the non-adaptation group.

## Data Availability

The datasets during and/or analyzed during the current study available from the corresponding author on reasonable request.
